# Circular RNA circ_SETD2 represses breast cancer progression via modulating the miR-155-5p/SCUBE2 axis

**DOI:** 10.1515/med-2020-0223

**Published:** 2020-09-30

**Authors:** Yuanyuan Shen, Mengmeng Zhang, Liangshan Da, Wei Huang, Congjun Zhang

**Affiliations:** Department of Oncology, High-tech District of the First Affiliated Hospital of Anhui Medical University, No.120 Wan Shui Road, Hefei, Anhui, 230022, China

**Keywords:** BC, circ_SETD2, miR-155-5p, SCUBE2

## Abstract

Breast cancer (BC) is the leading cause of cancer deaths in women worldwide. Circular RNA circ_SETD2 (circ_SETD2), also termed as hsa_circ_0065173, is reported to be abnormally expressed in BC. Nevertheless, the role and mechanism of circ_SETD2 in BC are unclear. Expression of circ_SETD2, miR-155-5p, and SCUBE2 mRNA was evaluated by quantitative real-time polymerase chain reaction. Cell cycle progression, proliferation, apoptosis, migration, and invasion were determined by flow cytometry, MTT, and transwell assays. The relationship between circ_SETD2 or SCUBE2 and miR-155-5p was verified through a dual-luciferase reporter assay. The role of circ_SETD2 in BC *in vivo* was confirmed by a xenograft assay. circ_SETD2 and SCUBE2 were downregulated, while miR-155-5p was upregulated in BC tissues and cells. Both circ_SETD2 and SCUBE2 elevation arrested cell cycle progression, inhibited cell proliferation, migration, and invasion, and accelerated cell apoptosis in BC cells. Moreover, circ_SETD2 upregulation repressed BC growth *in vivo*. Importantly, circ_SETD2 modulated SCUBE2 expression through competitively binding to miR-155-5p in BC cells. Also, the inhibitory impacts of circ_SETD2 enhancement on the malignant behavior of BC cells were restored by miR-155-5p overexpression. Besides, SCUBE2 silencing abolished miR-155-5p downregulation mediated effects on the malignant behavior of BC cells. Therefore, circ_SETD2 curbed BC progression via upregulating SCUBE2 via binding to miR-155-5p.

## 1 Introduction

Breast cancer (BC) is one of the most common malignant tumors in women, and it ranks second among the causes of cancer-related deaths among women worldwide [1]. Although great progress has been made in the treatment of BC, effective control of recurrence and metastasis remains a major obstacle [2,3]. Hence, understanding the molecular mechanism of BC progression is of great significance for exploring new therapeutic targets [4].

Circular RNAs (circRNAs) are characterized by a continuous covalent closed loop, without a 3′-poly A tail and a 5′-cap structure [5]. They exert an essential role in the diagnosis and development of human diseases, inducing cancers [6,7]. For instance, circRNA circ_102171 accelerated the advancement of papillary thyroid cancer by regulating the Wnt/β-catenin pathway in a CTNNBIP1-dependent method [8]. And circRNA circ_100290 regulated the miR-29b/CDK6 axis in oral squamous cell cancer cells, which could facilitate the progression of oral squamous cell cancer [9]. Circular RNA circ_SETD2 (circ_SETD2), located at chr3:47155365-47168153 with a length of 4644 bp, is produced by reverse splicing of the SET domain containing 2 (SETD2) gene. circ_SETD2 was revealed to be a differentially expressed circRNA in luminal A subtypes and triple-negative BC [10]. However, the role of circ_SETD2 in BC and its related molecular mechanisms are indistinct.

As small non-coding RNAs, microRNAs (miRNAs) mainly function as post-transcriptional regulators of target mRNAs [11]. miRNAs were demonstrated to play vital roles in cancer biologies, such as proliferation, invasion/metastasis, apoptosis, and angiogenesis [12]. It was reported that miRNA-155-5p (miR-155-5p) was connected with the progression of a range of tumors. miR-155-5p could expedite the advancement of cervical cancer [13] and hepatocellular cancer [14]. Besides, miR-155-5p was revealed to act as a tumor suppressor in gastric cancer [15]. Also, miR-155-5p antagonized the bufalin-mediated effect on the apoptosis of triple-negative BC cells [16]. However, the exact role of miR-155-5p in BC is unclear.

As a secreted and membrane-related multi-domain glycoprotein, signal peptide-CUB-epidermal growth factor-like domain-containing 2 (SCUBE2) plays vital roles in the advancement of tumors [17]. Reduced SCUBE2 expression was related to the prognosis and progression in colorectal cancer [18]. Also, SCUBE2 could repress cell migration, invasion, and proliferation in non-small cell lung cancer [19] and glioma cells [20]. Furthermore, SCUBE2 could curb BC cell invasion and migration [21]. However, whether SCUBE2 can be regulated by circ_SETD2 and miR-155-5p has never been reported.

Hence, we investigated the role of circ_SETD2 and SCUBE2 in BC. Moreover, we also explored the regulatory mechanism between circ_SETD2 and SCUBE2 in the advancement of BC. Our results indicated that circ_SETD2 repressed BC progression via modulating SCUBE2 expression via competitively binding to miR-155-5p.

## 2 Materials and methods

### 2.1 BC specimen collection

A total of 54 pairs of BC tissues and adjacent normal tissues were obtained from the The First Affiliated Hospital of Anhui Medical University. Patients who participated in this study did not receive radiotherapy and chemotherapy before undergoing surgical resection. This research was reviewed and approved by the Ethics Committee of The First Affiliated Hospital of Anhui Medical University, and all patients signed the informed consent forms.

### 2.2 Cell culture and transfection

Human normal mammary epithelial cells MCF-10A and BC cells (MCF-7 and MDA-MB-231) were bought from the American Type Culture Collection (Manassas, VA, USA). Based on a previously described method, the MCF-10A cells were cultured [22]. All cells were kept in a humidified atmosphere with 5% CO_2_ at 37°C. The MCF-7 and MDA-MB-231 cells were cultured in Dulbecco’s modified Eagle’s medium (Sigma, Louis, Missouri, MO, USA) supplemented with fetal bovine serum (FBS, 10%), streptomycin (100 µg/mL; Sigma), and penicillin (100U/mL; Sigma).

The sequence of circ_SETD2 or SCUBE2 was cloned into the pLCDH-ciR vector (Vector) (Greenseed Biotech, Guangzhou, China) or pcDNA3.1 vector (pcDNA) (Invitrogen, Carlsbad, CA, USA) to construct the overexpression vectors for circ_SETD2 and SCUBE2, respectively. Small interference RNA targeting SCUBE2 (si-SCUBE2) and negative control (si-NC) were obtained from RiboBio (Guangzhou, China). miRNA mimics and inhibitors targeting miR-155-5p (miR-155-5p and anti-miR-155-5p) and their negative controls (miR-NC and anti-miR-NC) were procured from RiboBio. The assigned oligonucleotides or vectors were transiently transfected into BC cells using Lipofectamine 3000 reagent (Invitrogen).

### 2.3 Quantitative real-time polymerase chain reaction (qPCR)

Total RNA of BC tissues and cells was extracted by using the TRIzol reagent (Invitrogen). For circ_SETD2, SETD2, and SCUBE2, a PrimeScript RT reagent Kit (Takara, Dalian, China) was applied to generate complementary DNA (cDNA). For miRNA (miR-155-5p and miR-619-3p), a First-Strand Synthesis Kit (Takara) was employed to generate cDNA. qRT-PCR was conducted through the SYBR Premix Ex Taq (Takara). The expression of circ_SETD2, SETD2, SCUBE2, miR-155-5p, and miR-619-3p was calculated by the 2^-ΔΔCt^ method, and glyceraldehyde-3-phosphate dehydrogenase (GAPDH) or U6 small nuclear RNA (snRNA) was utilized as the internal control. The primers used in this study were as follows: circ_SETD2: (F: 5′-CTTGAGAGCTGCCAAAGACCT-3′ and R: 5′-TTGGTGCCTTTGGGCAAAAATCC-3′); SETD2: (F: 5′-GGCCAGCCTTGTGCTATGTT-3′ and R: 5′-CTTTTAGGGAACACACATGCCA-3′); SCUBE2 (F: 5′-CCCCCAAGCGCCGCATCCTGA-3′ and R: 5′-TATTGAGTGGCACGTGGGCTGAGT-3′); GAPDH: (F: 5′-GACTCCACTCACGGCAAATTCA-3′ and R: 5′-TCGCTCCTGGAAGATGGTGAT-3′); miR-155-5p: (F: 5′-UAAUACCGUCUUAAAACCGU-3′ and R: 5′-UUCUGGGAACGUGAAACCT-3′); and U6 snRNA (F: 5′-GCTCGCTTCGGCAGCACA-3′ and R: 5′-GAGGTATTCGCACCAGAGGA-3′).

### 2.4 RNase R and actinomycin D treatment

Total RNA of BC cells was digested with RNase R (3 U/μg; Epicentre Technologies, Madison, WI, USA) at 37°C for 15 min. Actinomycin D (2 mg/mL; Sigma) was supplemented in the cell medium to block transcription. After treatment with RNase R and actinomycin D, the expression of circ_SETD2 and SETD2 mRNA was examined by qRT-PCR.

### 2.5 Propidium iodide (PI) cytometry assay

After transfection for 48 h, the cells were fixed with ethanol (70%) at −20°C overnight. Then, the fixed cells were incubated with RNase A (0.1 mg/mL; Beyotime, Shanghai, China) and PI (50 µg/mL; Sigma) at −20°C for 30 min. A FACScan flow cytometer (BD Biosciences, San Jose, CA, USA) was utilized for the evaluation of the distribution of the cell cycle.

### 2.6 Western blot analysis

Western blot analysis was executed as previously described [23]. BC tissues and cells were lysed in lysis buffer (Beyotime). The bands were assessed through EZ-ECL chemiluminescence (Biological Industries, Beit-Haemek, Israel) and visualized using an ImmunoStar LD (Wako Pure Chemical, Osaka, Japan). The primary antibodies were as follows: anti-cyclin D1 (ab16663, 1:1,000; Abcam, Cambridge, MA, USA), anti-Cell Cycle Dependent Kinase 4 (CDK4) (ab108357, 1:500; Abcam), anti-GAPDH (ab128915, 1:5,000; Abcam), and anti-SCUBE2 (1:500; Santa Cruz Biotechnology, Santa Cruz, CA, USA). Goat anti-rabbit (ab97051, 1:10,000; Abcam) or mouse (ab205719, 1:2,000; Abcam) immunoglobulin G (IgG) was used as the secondary antibody. GAPDH was regarded as a loading control.

### 2.7 Cell proliferation assay

The proliferation of transfected BC cells was analyzed by a 3-[4,5-dimethylthiazol-2-yl]-2,5-diphenyltetrazolium bromide (MTT) assay. In short, transfected BC cells were seeded into 96-well plates (2.5 × 10^3^ cells per well) and cultured for 24, 48, and 72 h. Afterward, 20 μL of MTT (5 mg/mL; Sigma) was added to each well. After incubating for 4 h, dimethyl sulfoxide (150 μL) was supplemented to each well for the dissolution of the formazan crystals. A microplate absorbance reader (Molecular Devices LLC, Sunnyvale, CA, USA) was utilized to analyze the color reaction at 490 nm.

### 2.8 Flow cytometry assay

An annexin V-fluorescein isothiocyanate (FITC)/PI apoptosis detection kit (Sigma) was employed to determine the apoptosis of transfected BC cells. In short, transfected BC cells were resuspended in binding buffer. Following this, the cells were incubated with annexin V-FITC (5 µL) and PI (10 µL) at 4°C for 15 min. The sample was assessed through a FACScan flow cytometer (BD Biosciences).

### 2.9 Transwell assay

In the migration assay, transfected BC cells (1 × 10^5^) were seeded into the upper chamber of a transwell chamber (8 µm; Corning Costar, Corning, NY, USA), and the culture medium with FBS (10%) was supplemented to the lower chamber of the transwell chamber. The cells on the lower membrane were fixed using paraformaldehyde (4%; Beyotime) and stained using crystal violet (0.1%; Sigma) after culture for 24 h. The same method was applied to assess the invasion ability of transfected BC cells, but the upper chamber of the transwell chamber was covered using a matrigel matrix (BD Biosciences). A light microscope (Olympus) was used to observe the migrating and invading cells.

### 2.10 Dual-luciferase reporter assay

The binding sites between circ_SETD2 or SCUBE2 and miR-155-5p were predicted using CircInteractome or miRDB databases. The wild type (WT) and mutant (MUT) luciferase reporter vectors of circ_SETD2 were constructed by inserting the sequences of circ_SETD2 and mutant circ_SETD2 (within the binding sites for miR-155-5p) into the pGL3-control vector (Promega, Madison, WI, USA), respectively. And the same method was used to construct the luciferase reporter vectors for SCUBE2. The luciferase reporter vectors and miR-NC or miR-155-5p were cotransfected into BC cells using Lipofectamine 3000 reagent. A dual-luciferase reporter assay kit (Promega) was employed to determine the luciferase intensities of the luciferase reporter vectors.

### 2.11 Xenograft assay

The animal experiment was approved by the Ethics Committee of The First Affiliated Hospital of Anhui Medical University. Ten BALB/c nude mice (4–6 weeks old) were obtained from the Shanghai Experimental Animal Center (Shanghai, China) and housed in a specific pathogen-free room. Briefly, MCF-7 cells with vector or stable lentivirus-mediated circ_SETD2 (RiboBio) were resuspended in phosphate buffer solution (200 µL) and then were subcutaneously injected into the dorsal side of the nude mice (5 mice per group). Tumor volume was measured using a digital caliper every 5 days from day 10. The mice were euthanized to harvest their tumor tissues for subsequent studies on day 30 after injection. Tumor volume was calculated as follows: volume = (length × width^2^)/2.

### 2.12 Statistical analysis

The experiments in the *in vitro* research were repeated at least three times. SPSS 19.0 software (IBM Corporation, Armonk, NY, USA) was utilized for statistical analysis. The Kolmogorov–Smirnov test was applied to determine the normal distribution of the data in each group. The homogeneity of the variances was assessed via the *F*-test. The differences between two groups or among more groups were evaluated with Student’s *t* test or one-way analysis of variance (ANOVA) followed by Tukey’s *post hoc* test. The correlation was evaluated via Pearson’s correlation analysis. Data were exhibited as mean ± standard deviation. Differences were deemed significant if *P* < 0.05.

## 3 Results

### 3.1 Expression and characteristics of circ_SETD2 in BC

**Figure 1:**
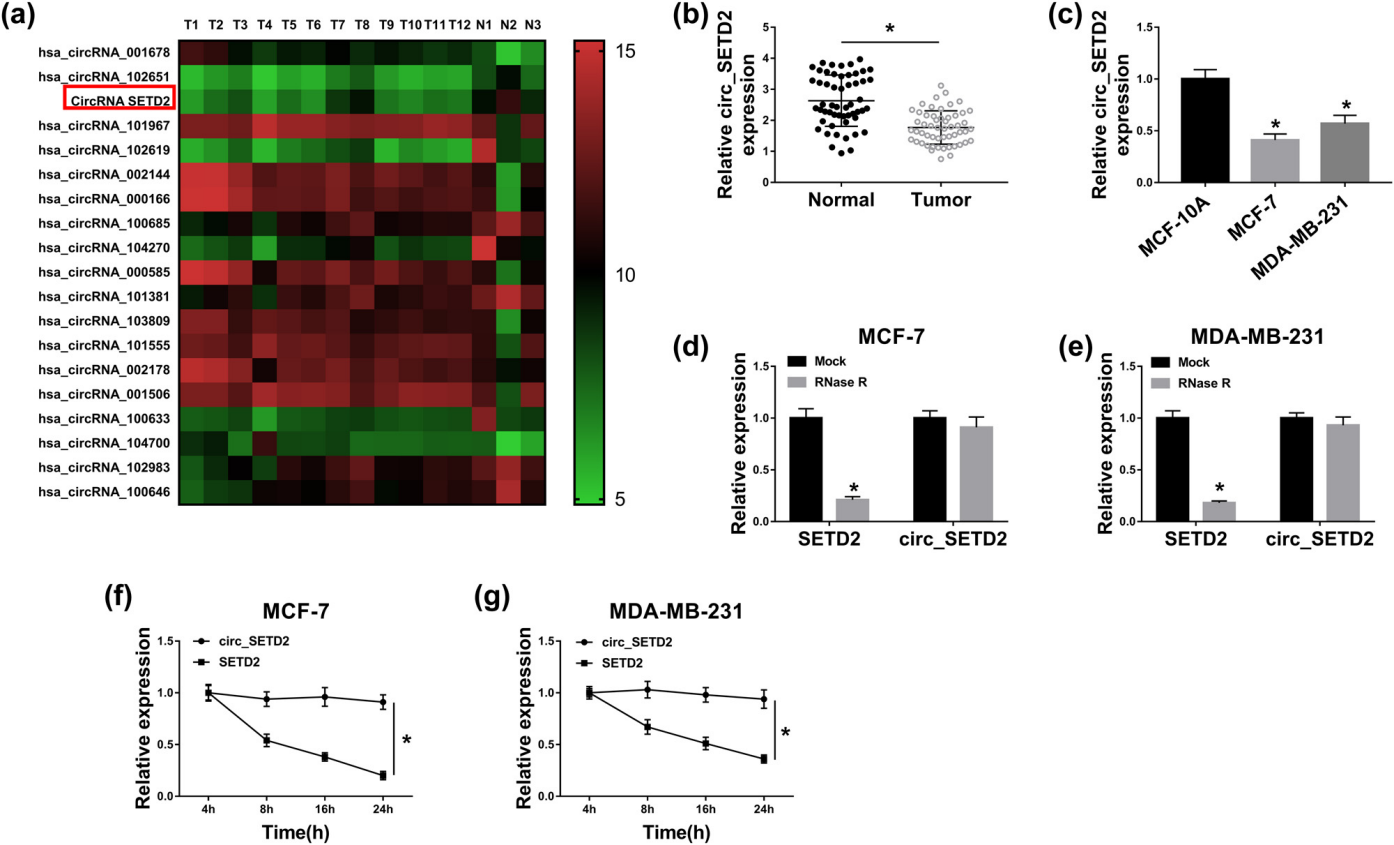
Identification and expression of circ_SETD2 in BC tissues and cells. (a) The expression of circ_SETD2 in BC tissues in comparison to normal tissues was analyzed by using the GEO dataset GSE101124. (b and c) Relative circ_SETD2 expression in BC tissues and adjacent normal tissues, as well as BC cells (MCF-7 and MDA-MB-231) and MCF-10A cells was examined by qRT-PCR. (d and e) The levels of circ_SETD2 and SETD2 in total RNA of MCF-7 and MDA-MB-231 cells with or without RNase R treatment were analyzed by qRT-PCR. (f and g) The levels of circ_SETD2 and SETD2 in MCF-7 and MDA-MB-231 cells treated with actinomycin D for different times were assessed with qRT-PCR. The data are presented as mean ± standard deviation; paired Student’s *t* test was utilized to compare the differences between BC tissues and adjacent normal tissues; one-way ANOVA followed by Tukey’s *post hoc* test was utilized to compare the differences among groups; the experiments were repeated three times independently. *P < 0.05.

To survey the abnormal circRNAs involved in BC advancement, we first analyzed the microarray data from Gene Expression Omnibus (GEO) datasets. And the results exhibited that circ_SETD2 was overtly downregulated in BC tissues in the GSE101124 dataset (Figure 1a). Then, we detected circ_SETD2 expression in 54 pairs of BC tissues and adjacent normal tissues to verify the microarray analysis results. qRT-PCR displayed that the expression of circ_SETD2 was distinctly reduced in BC tissues in comparison to the adjacent normal tissues (Figure 1b). Moreover, circ_SETD2 expression was markedly decreased in BC cells (MCF-7 and MDA-MB-231) in contrast to the MCF-10A cells (Figure 1c). Subsequently, total RNA from MCF-7 and MDA-MB-231 cells was treated with RNase R to assess the resistance of circ_SETD2. We observed that the linear gene SETD2 was digested by RNase R, while the circ_SETD2 was not affected (Figure 1d and e). Additionally, with the extension of time, the linear gene SETD2 was gradually degraded by actinomycin D, but the circ_SETD2 did not change (Figure 1f and g). Therefore, these results indicated that low circ_SETD2 expression might be related to BC progression.

### 3.2 Elevated circ_SETD2 expression blocked cell cycle progression, repressed cell proliferation, migration, invasion, and accelerated cell apoptosis in BC cells

**Figure 2:**
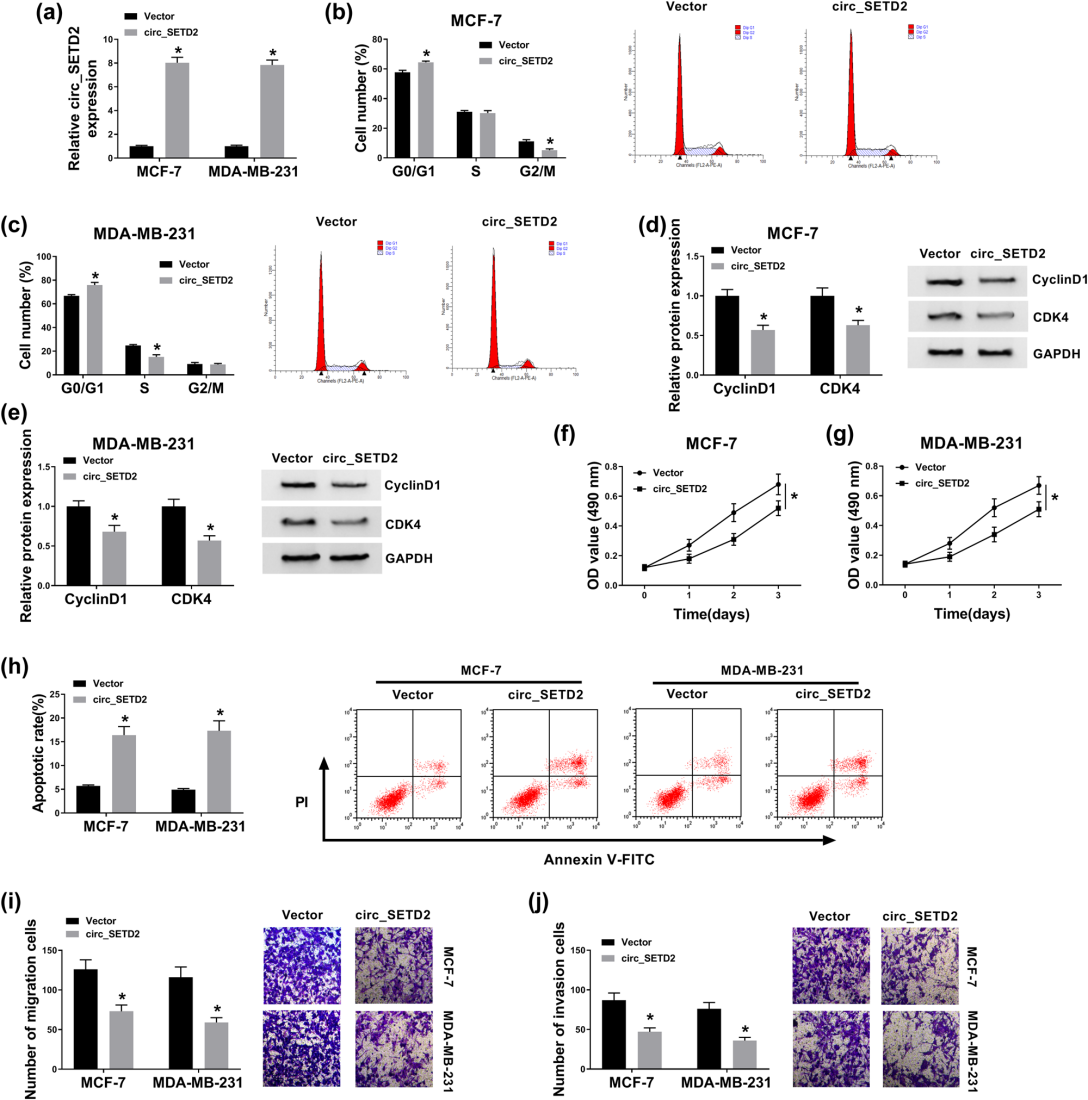
Effects of circ_SETD2 introduction on cell cycle, proliferation, apoptosis, migration, and invasion of BC cells. (a) qRT-PCR was performed to analyze the expression of circ_SETD2 in MCF-7 and MDA-MB-231 cells transfected with circ_SETD2 or vector. (b and c) Influence of circ_SETD2 overexpression on cell cycle progression of MCF-7 and MDA-MB-231 cells was assessed by PI cytometry assay. (d and e) Impacts of circ_SETD2 upregulation on the expression of cyclin D1 and CDK4 proteins of MCF-7 and MDA-MB-231 cells were evaluated by western blot analysis. (f–j) Effects of circ_SETD2 elevation on cell proliferation, apoptosis, migration, and invasion in MCF-7 and MDA-MB-231 cells were determined with the MTT (f and g), flow cytometry (h), and transwell assays (i and j). The data are presented as mean ± standard deviation; one-way ANOVA followed by Tukey’s *post hoc* test was utilized to compare the differences among groups; the experiments were repeated three times. *P < 0.05.

In view of the reduced expression of circ_SETD2 in BC tissues and cells, we further explored the role of circ_SETD2 in BC progression via a gain-of-function experiment. qRT-PCR exhibited that circ_SETD2 expression was enhanced in MCF-7 and MDA-MB-231 cells transfected with circ_SETD2 compared to the control group (Figure 2a). Subsequently, the impacts of circ_SETD2 overexpression on cell cycle progression, proliferation, migration, invasion, and apoptosis of BC cells were explored. The PI cytometry assay for cell cycle revealed that circ_SETD2 enhancement boosted the number of MCF-7 and MDA-MB-231 cells in the G0/G1 stage of the cell cycle (Figure 2b and c). Moreover, the cycle-associated proteins (cyclin D1 and CDK4) were detected by western blot analysis. The results displayed that the protein levels of cyclin D1 and CDK4 were strikingly reduced in circ_SETD2-upregulated MCF-7 and MDA-MB-231 cells (Figure 2d and e). The MTT assay manifested that the proliferation of MCF-7 and MDA-MB-231 cells was repressed by circ_SETD2 elevation (Figure 2f and g). Also, the flow cytometry assay for cell apoptosis exhibited that upregulated circ_SETD2 expression increased the cell apoptotic rate in MCF-7 and MDA-MB-231 cells (Figure 2h). The transwell assay presented that circ_SETD2 overexpression inhibited the migration and invasion capacities of MCF-7 and MDA-MB-231 cells (Figure 2i and j). Together, these findings revealed that circ_SETD2 overexpression could suppress malignant behavior in BC cells.

### 3.3 Enhancement of SCUBE2 arrested cell cycle progression, constrained cell proliferation, migration, and invasion, and induced cell apoptosis in BC cells

**Figure 3:**
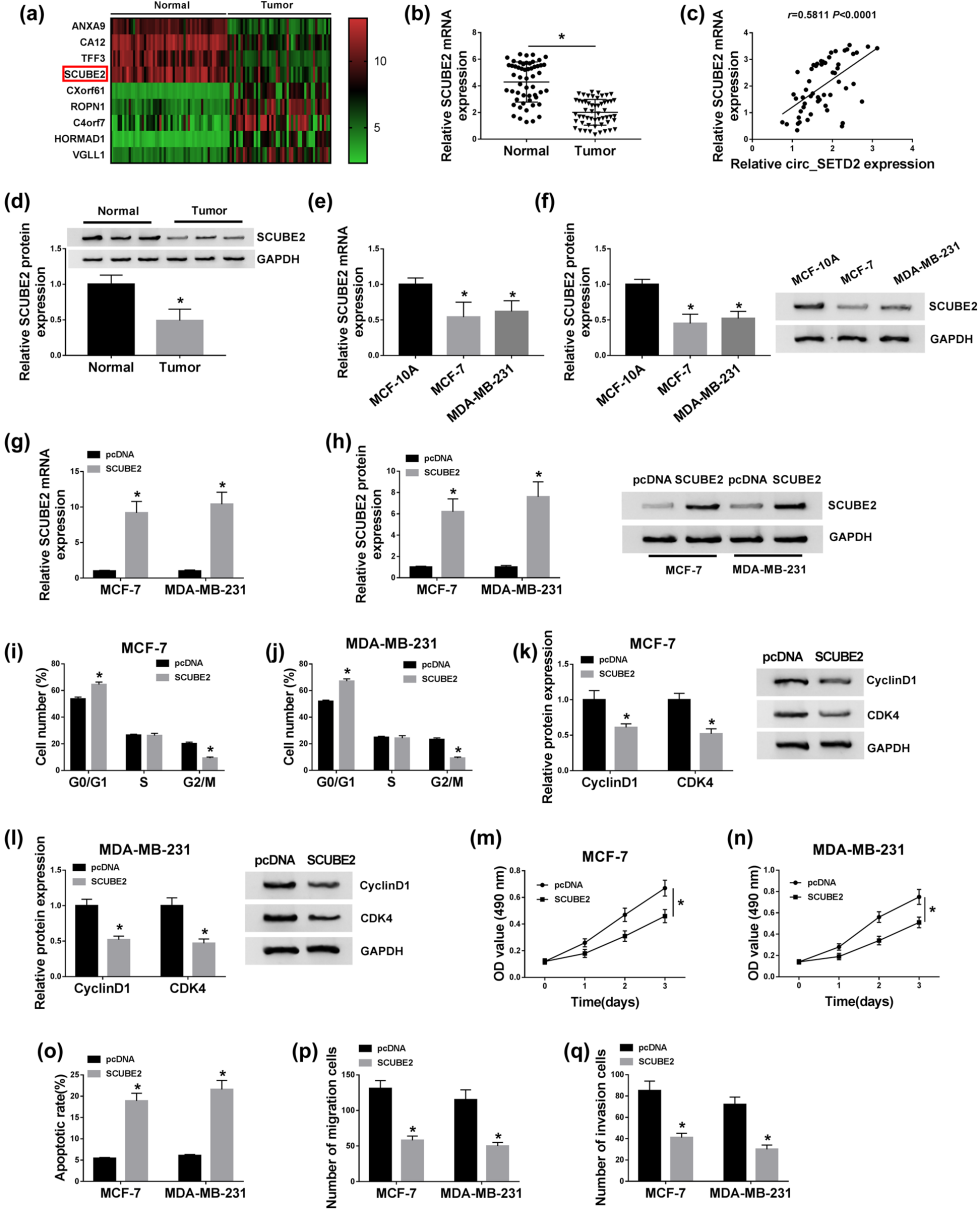
Influence of SCUBE2 introduction on cell cycle progression, proliferation, apoptosis, migration, and invasion of BC cells. (a) The expression of SCUBE2 in BC tissues compared with normal tissues was assessed through using the GEO dataset GSE62931. (b) The mRNA level of SCUBE2 in BC tissues and adjacent normal tissues was examined with qRT-PCR. (c) Pearson’s correlation analysis was used to evaluate the correlation between SCUBE2 and circ_SETD2 in BC tissues. (d) Expression of SCUBE2 protein in BC tissues and adjacent normal tissues was detected by western blot analysis. (e–h) The mRNA and protein levels of SCUBE2 in MCF-7 and MDA-MB-231 cells, as well as MCF-7 and MDA-MB-231 cells transfected with SCUBE2 or pcDNA were assessed by qRT-PCR or western blot analysis. (i and j) The effect of SCUBE2 upregulation on cell cycle progression of MCF-7 and MDA-MB-231 cells was assessed by PI cytometry assay. (k and l) The expression of cyclin D1 and CDK4 proteins in MCF-7 and MDA-MB-231 cells transfected with SCUBE2 or pcDNA was detected through western blot analysis. (m–q) Impacts of SCUBE2 elevation on cell proliferation, apoptosis, migration, and invasion in MCF-7 and MDA-MB-231 cells were analyzed by MTT (m and n), flow cytometry (o), and transwell assays (p and q). The data are presented as mean ± standard deviation; paired Student’s *t* test was utilized to compare the differences between BC tissues and adjacent normal tissues; one-way ANOVA followed by Tukey’s *post hoc* test was utilized to compare the differences among groups; the experiments were repeated three times. *P < 0.05.

Subsequently, we used mRNA microarray data in the GEO dataset to screen genes with significant differences in BC tissues and normal tissues. As exhibited in Figure 3a, ANXA9, CA12, TFF3, and SCUBE2 genes were remarkably downregulated in BC tissues compared to the normal tissues in the GSE62931 dataset. And circ_SETD2 overexpression overtly increased the mRNA and protein levels of SCUBE2 in both MCF-7 and MDA-MB-231 cells (Figure S1a–d). Therefore, we selected SCUBE2 as the downstream gene for research. We observed that the expression of SCUBE2 mRNA was conspicuously decreased in BC tissues when compared to the adjacent normal tissues (Figure 3b). Moreover, the expressions of SCUBE2 and circ_SETD2 in BC tissues were positively correlated (Figure 3c). Furthermore, SCUBE2 protein was also reduced in BC tissues in contrast to the adjacent normal tissues (Figure 3d). Consistently, the mRNA and protein levels of SCUBE2 were reduced in MCF-7 and MDA-MB-231 cells (Figure 3e and f). We also observed that SCUBE2 mRNA and protein were upregulated in MCF-7 and MDA-MB-231 cells transfected with SCUBE2 (Figure 3g and h). Moreover, the cell cycle of MCF-7 and MDA-MB-231 cells was arrested in the G0/G1 stage by SCUBE2 overexpression (Figure 3i and j). And the expression of cyclin D1 and CDK4 proteins was repressed in SCUBE2-upregulated MCF-7 and MDA-MB-231 cells (Figure 3k and l). Besides, augmented SCUBE2 expression curbed cell proliferation and promoted cell apoptosis in MCF-7 and MDA-MB-231 cells (Figure 3m–o). In addition, SCUBE2 introduction blocked the migration and invasion of MCF-7 and MDA-MB-231 cells (Figure 3p and q). In all, SCUBE2 upregulation could curb the malignant behavior of BC cells.

### 3.4 circ_SETD2 acted as a sponge for miR-155-5p in BC cells, and miR-155-5p targeted SCUBE2

**Figure 4:**
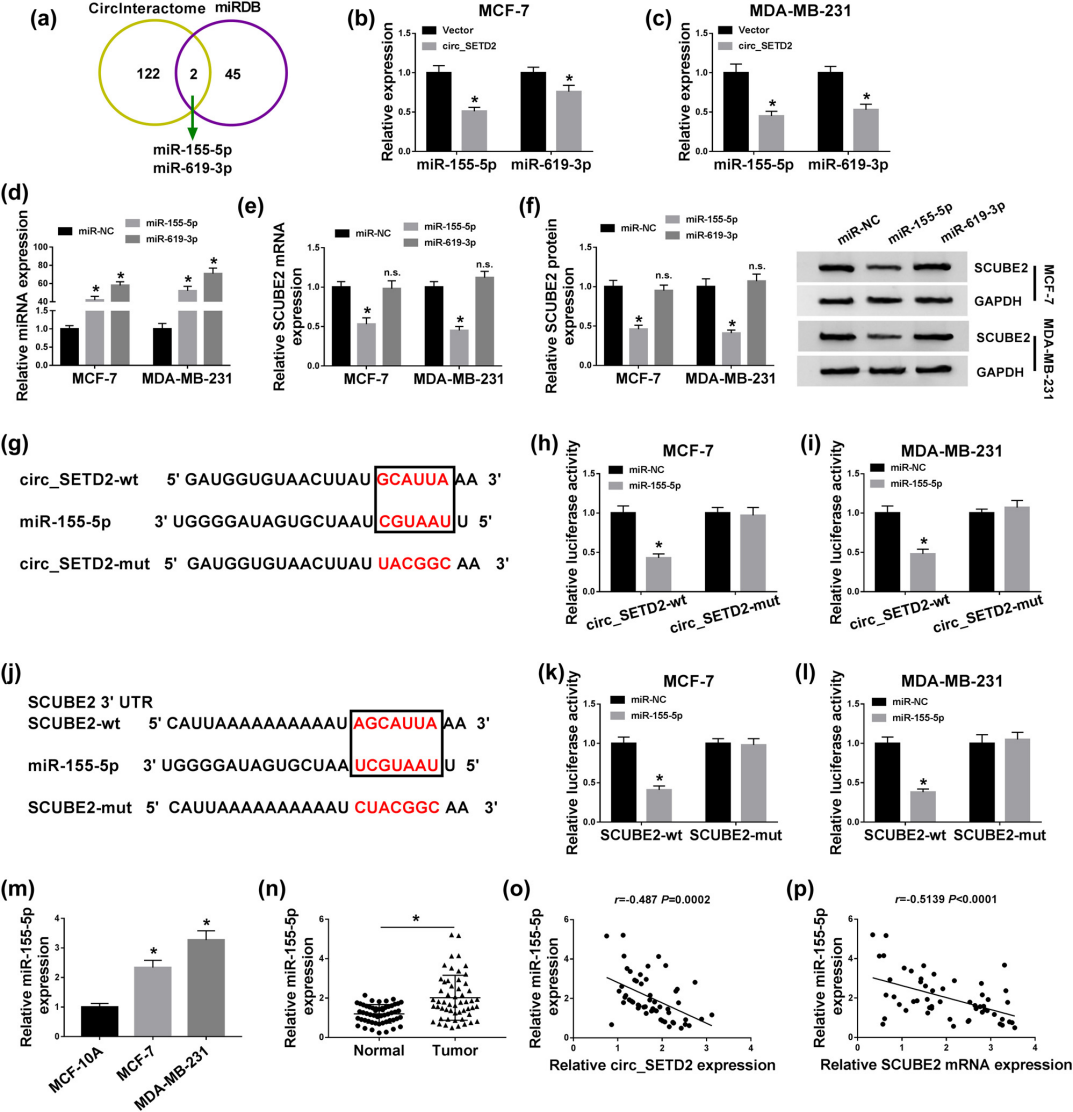
miR-155-5p and SCUBE2 served as a target for circ_SETD2 or miR-155-5p in BC cells. (a) The relationship between circ_SETD2 or SCUBE2 and miR-155p-5p or miR-619-3p was predicted using the CircInteractome or miRDB database. (b and c) Effect of circ_SETD2 overexpression on miR-155-5p or miR-619-3p expression in MCF-7 and MDA-MB-231 cells was determined by qRT-PCR. (d) The transfection efficiency of miR-155-5p or miR-619-3p in MCF-7 and MDA-MB-231 cells was assessed by qRT-PCR. (e and f) The impacts of miR-155-5p or miR-619-3p enhancement on the expression of SCUBE2 mRNA and protein were evaluated by qRT-PCR or western blot analysis. (g) The binding sites of miR-155-5p in circ_SETD2. (h and i) Dual luciferase reporter assay was executed to assess the luciferase intensity of the circ_SETD2-wt or circ_SETD2-mut reporter vectors in MCF-7 and MDA-MB-231 cells transfected with miR-155-5p or miR-NC. (j) The binding sites of SCUBE2 in miR-155-5p. (k and l) Dual luciferase reporter assay was carried out to determine the luciferase activity of the SCUBE2-wt or SCUBE2-mut reporter vectors in MCF-7 and MDA-MB-231 cells transfected with miR-155-5p or miR-NC. (m and n) The expression of miR-155-5p in BC cells (MCF-7 and MDA-MB-231) and tissues was examined by qRT-PCR. (o and p) The correlation between miR-155-5p and circ_SETD2 or SCUBE2 in BC tissues was determined by Pearson’s correlation analysis. The data are presented as mean ± standard deviation; paired Student’s *t* test was utilized to compare the differences between BC tissues and adjacent normal tissues; one-way ANOVA followed by Tukey’s *post hoc* test was utilized to compare the differences among groups; the experiments were repeated three times. *P < 0.05.

To explore the regulatory mechanism between circ_SETD2 and SCUBE2 in BC, we predicted the miRNAs that could be combined with circ_SETD2 and SCUBE2 through the CircInteractome or miRDB database, respectively. The results displayed that miR-155-5p and miR-619-3p had the possible binding sites for circ_SETD2 and SCUBE2 in the CircInteractome and miRDB databases (Figure 4a). Moreover, circ_SETD2 enhancement could inhibit the expression of miR-155-5p and miR-619-3p in MCF-7 and MDA-MB-231 cells (Figure 4b and c). And the expression of miR-155-5p and miR-619-3p was elevated in MCF-7 and MDA-MB-231 cells transfected with miR-155-5p or miR-619-3p compared to the miR-NC (Figure 4d). Furthermore, miR-155-5p overexpression could repress the expression of SCUBE2 mRNA and protein in MCF-7 and MDA-MB-231 cells, but the expression of SCUBE2 mRNA and protein was not affected by miR-619-3p upregulation (Figure 4e and f). So, we selected miR-155-5p for further studies. The binding sites between circ_SETD2 and miR-155-5p are presented in Figure 4g. The dual luciferase reporter assay presented that miR-155-5p upregulation repressed the luciferase activity of the circ_SETD2-wt reporter vectors in MCF-7 and MDA-MB-231 cells, while the luciferase activity of the circ_SETD2-mut reporter vectors was not remarkably changed (Figure 4h and i). In addition, we further verified the relationship between miR-155-5p and SCUBE2. The binding sites of SCUBE2 in miR-155-5p are exhibited in Figure 4j. And the luciferase intensity of the SCUBE2-wt reporter vectors in miR-155-5p-enhanced MCF-7 and MDA-MB-231 cells was strikingly repressed, but there was no prominent difference in the luciferase intensity of the SCUBE2-mut reporter vectors (Figure 4k and l). qRT-PCR showed that miR-155-5p expression was obviously boosted in BC cells (MCF-7 and MDA-MB-231) and tissues (Figure 4m and n). Pearson’s correlation analysis suggested that the expression of miR-155-5p in BC tissues was negatively correlated with circ_SETD2 and SCUBE2, respectively (Figure 4o and p). In all, these results indicated that miR-155-5p acted as a target for circ_SETD2, and miR-155-5p targeted SCUBE2 in BC cells.

### 3.5 miR-155-5p upregulation reversed circ_SETD2 introduction-mediated effects on cell cycle progression, proliferation, apoptosis, migration, and invasion of BC cells

**Figure 5:**
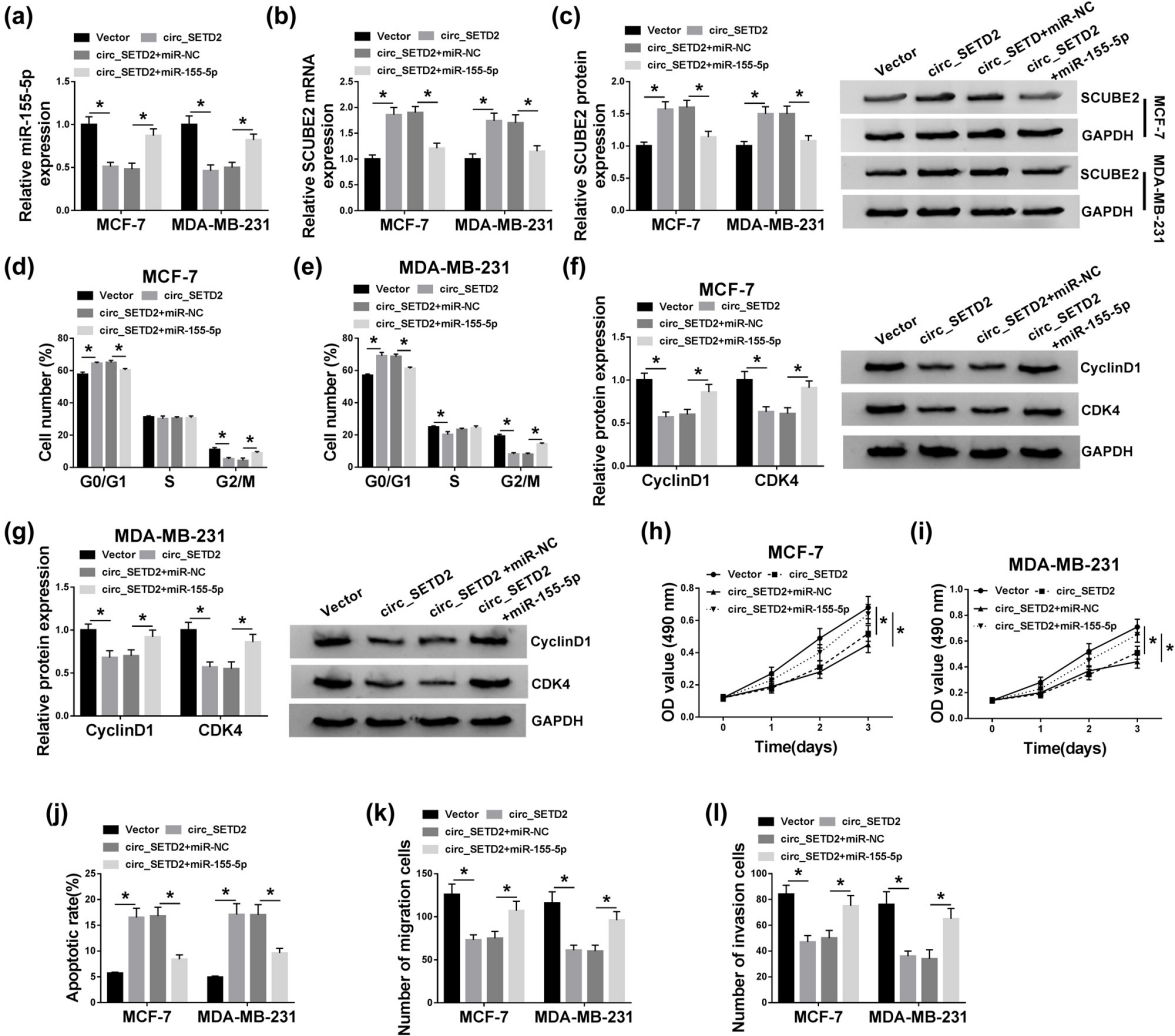
circ_SETD2 regulated the progression of BC via miR-155-5p. (a–c) qRT-PCR and western blot analysis were employed to assess the effects of miR-155-5p overexpression on the expression of miR-155-5p and the expression of SCUBE2 mRNA and protein in MCF-7 and MDA-MB-231 cells mediated by circ_SETD2 enhancement. (d and e) PI cytometry assay was performed to analyze the influence of miR-155-5p overexpression on cell cycle progression in MCF-7 and MDA-MB-231 cells caused by circ_SETD2 elevation. (f and g) Effects of miR-155-5p overexpression on circ_SETD2 elevation-mediated the expression of cyclin D1 and CDK4 proteins were determined by western blot analysis. (h–l) Influence of miR-155-5p introduction on circ_SETD2 overexpression-mediated proliferation, apoptosis, migration, and invasion of MCF-7 and MDA-MB-231 cells was evaluated by MTT (h and i), flow cytometry (j), and transwell assays (k and l). The data are presented as mean ± standard deviation; one-way ANOVA followed by Tukey’s *post hoc* test was utilized to compare the differences among groups; the experiments were repeated three times. *P < 0.05.

Given that miR-155-5p was a target of circ_SETD2, we further explored whether the influence of circ_SETD2 on BC progression was associated with miR-155-5p. Results presented that circ_SETD2 introduction could repress miR-155-5p expression in MCF-7 and MDA-MB-231 cells and boost SCUBE2 mRNA and protein expression, while this impact was abolished by miR-155-5p enhancement (Figure 5a–c). Moreover, the arrest of the cell cycle of circ_SETD2-enhanced MCF-7 and MDA-MB-231 cells was overturned by miR-155-5p upregulation (Figure 5d and e). And the repressive influence of circ_SETD2 overexpression on cyclin D1 and CDK4 protein expression was restored by miR-155-5p introduction (Figure 5f and g). Also, both the repression of proliferation and the acceleration of apoptosis of MCF-7 and MDA-MB-231 cells mediated by circ_SETD2 augmentation were recovered by miR-155-5p introduction (Figure 5h and j). Besides, augmented miR-155-5p expression restored the inhibitory effects on the migration and invasion of MCF-7 and MDA-MB-231 cells caused by circ_SETD2 overexpression (Figure 5k and l). These results revealed that circ_SETD2 modulated BC progression through miR-155-5p.

### 3.6 Inhibition of SCUBE2 reversed the influence of miR-155-5p silencing on cell cycle progression, proliferation, apoptosis, migration, and invasion of BC cells

**Figure 6:**
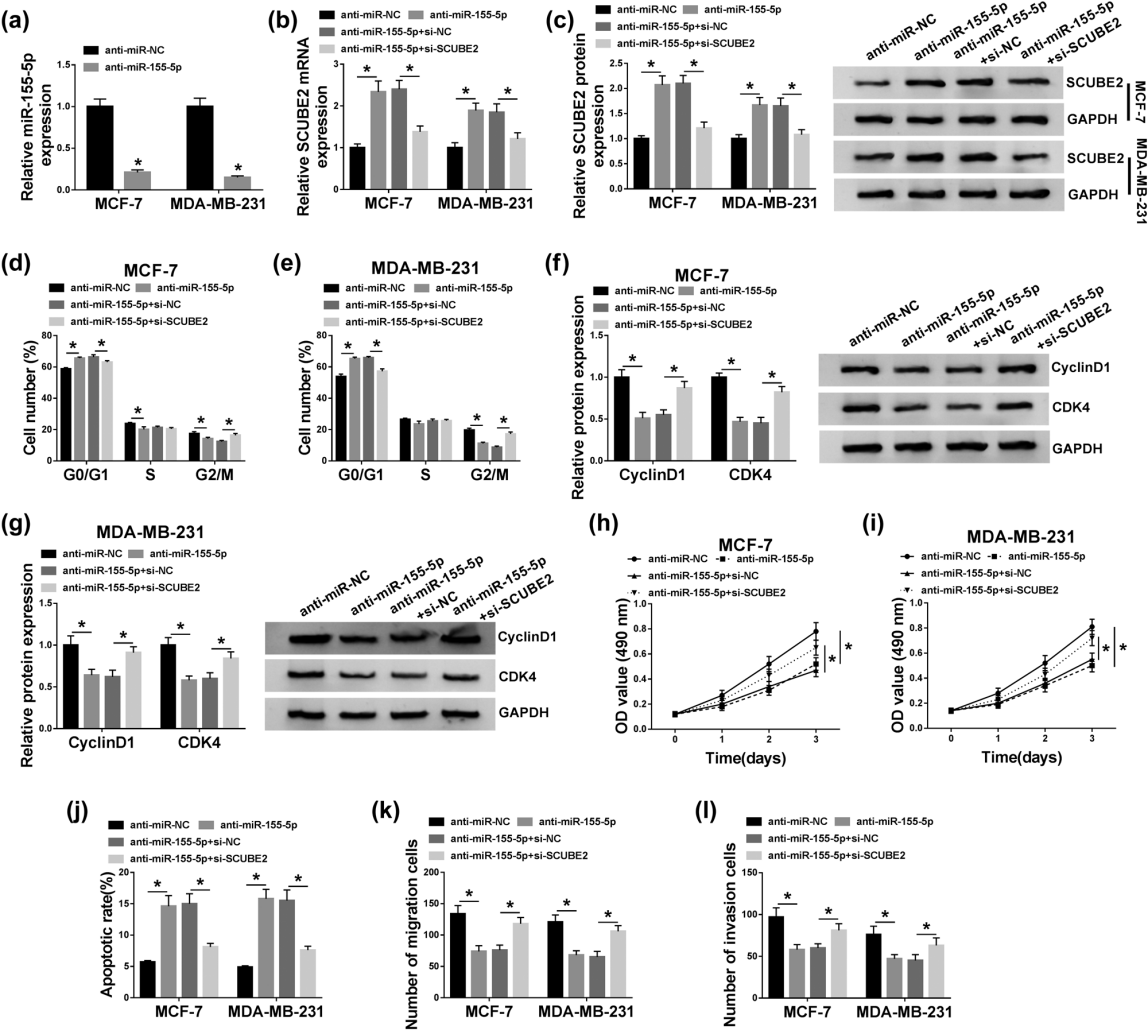
miR-155-5p played its role in BC cells via SCUBE2. (a) The expression of miR-155-5p in MCF-7 and MDA-MB-231 cells transfected with anti-miR-155-5p or anti-miR-NC was examined by qRT-PCR. (b–l) MCF-7 and MDA-MB-231 cells were transfected with anti-miR-NC, anti-miR-155-5p, anti-miR-155-5p + si-SCUBE2, or anti-miR-155-5p + si-NC. (b and c) The expression of miR-155-5p and SCUBE2 mRNA and protein in MCF-7 and MDA-MB-231 cells was detected by qRT-PCR or western blot analysis. (d and e) The cell cycle progression of MCF-7 and MDA-MB-231 cells was assessed via PI cytometry assay. (f and g) The protein levels of cyclin D1 and CDK4 in MCF-7 and MDA-MB-231 cells were examined through western blot analysis. (h–l) The proliferation, apoptosis, migration, and invasion of MCF-7 and MDA-MB-231 cells were determined by MTT (h and i), flow cytometry (j), and transwell assays (k and l). The data are presented as mean ± standard deviation; one-way ANOVA followed by Tukey’s *post hoc* test was utilized to compare the differences among groups; the experiments were repeated three times. *P < 0.05.

Next, we further surveyed whether miR-155-5p regulates the progression of BC via SCUBE2. Results presented that miR-155-5p expression was repressed in MCF-7 and MDA-MB-231 cells transfected with anti-miR-155-5p compared to the negative control (Figure 6a). Also, the downregulation of miR-155-5p promoted the expression of SCUBE2 mRNA and protein in MCF-7 and MDA-MB-231 cells, while this effect was recovered by SCUBE2 inhibition (Figure 6b and c). Furthermore, SCUBE2 depletion overturned the repressive influence of miR-155-5p silencing on cell cycle progression of MCF-7 and MDA-MB-231 cells (Figure 6d and e). And the downregulation of cyclin D1 and CDK4 proteins in MCF-7 and MDA-MB-231 cells caused by miR-155-5p depletion was reversed by SCUBE2 silencing (Figure 6f and g). Besides, reduced SCUBE2 expression abolished the inhibition of proliferation and the promotion of apoptosis of MCF-7 and MDA-MB-231 cells mediated by miR-155-5p suppression (Figure 6h–j). Additionally, the suppressive effects of miR-155-5p downregulation on cell migration and invasion in MCF-7 and MDA-MB-231 cells were restored by SCUBE2 exhaustion (Figure 6k and l). In short, miR-155-5p regulated BC advancement via SCUBE2.

### 3.7 circ_SETD2 upregulation repressed BC growth *in vivo*

**Figure 7:**
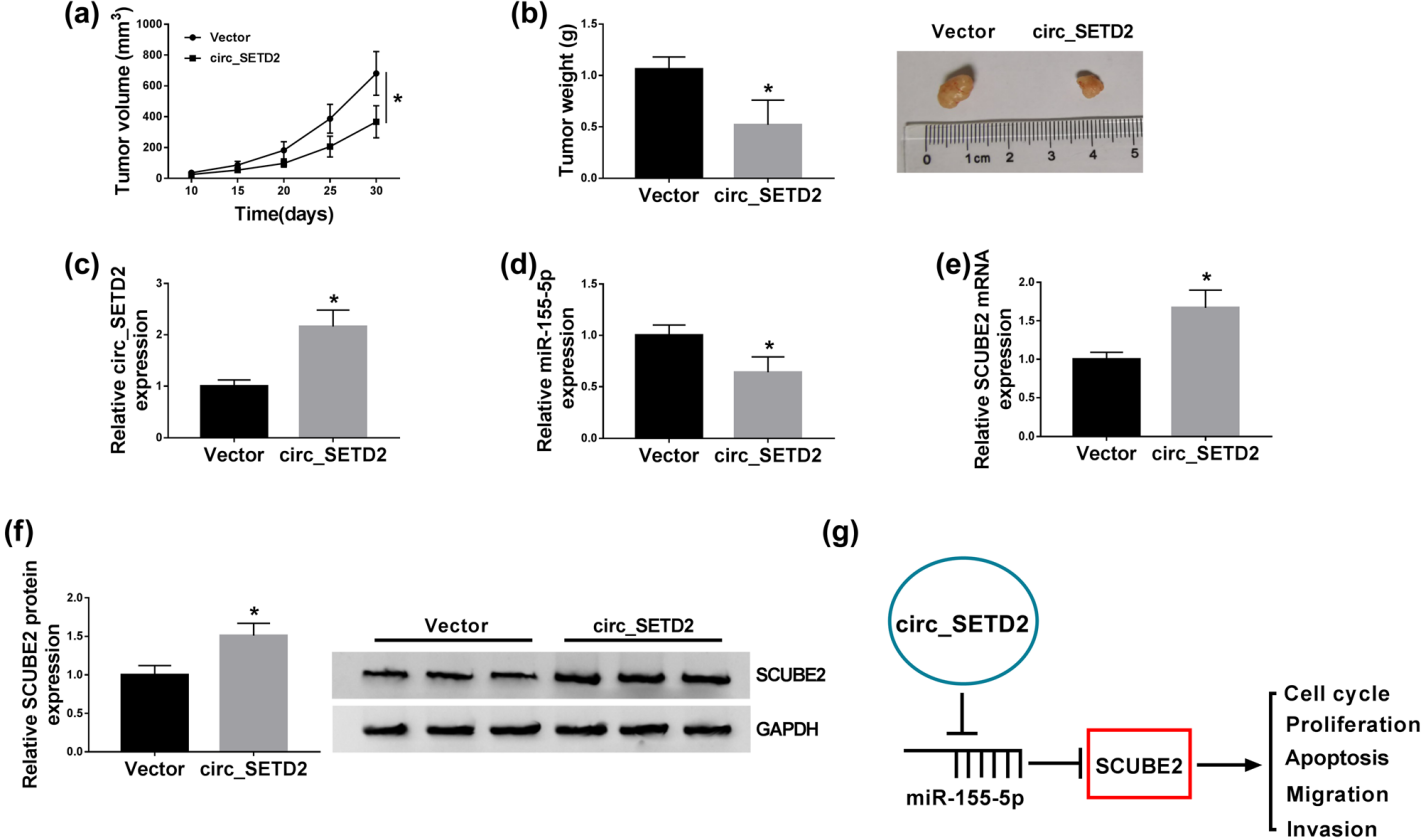
circ_SETD2 overexpression repressed BC growth *in vivo*. (a) From day 10, tumor volume was measured every 5 days until day 30. (b) Tumor weight was assessed on day 30 after injection. (c and d) The expression of circ_SETD2 and miR-155-5p in tumor tissues of the circ_SETD2 and vector groups was analyzed by qRT-PCR. (e and f) The expression of SCUBE2 mRNA and protein in tumor tissues of the circ_SETD2 and vector groups was examined with qRT-PCR or western blot analysis. (g) Schematic diagram of the mechanism of circ_SETD2 involved in BC advancement. The data are presented as mean ± standard deviation; paired Student’s *t* test was utilized to compare the differences between the circ_SETD2 and vector groups; the experiments *in vivo* were repeated three times. *P < 0.05.

We further verified the role of circ_SETD2 in BC *in vivo* through a xenograft assay. The results exhibited that circ_SETD2 overexpression repressed tumor growth (reduced tumor volume and weight) compared to the vector group (Figure 7a and b). Furthermore, circ_SETD2 expression was overtly elevated and miR-155-5p expression was markedly reduced in tumor tissues of the circ_SETD2 group when compared to the vector group (Figure 7c and d). Also, SCUBE2 mRNA and protein were prominently upregulated in tumor tissues of the circ_SETD2 group compared to that in the vector group (Figure 7e and f). Therefore, circ_SETD2 regulated the malignancy of BC cells via regulating SCUBE2 expression via competitively binding to miR-155-5p (Figure 7g). These data indicated that circ_SETD2 repressed BC growth *in vivo* through upregulating SCUBE2 via miR-155-5p.

## 4 Discussion

Recently, circRNAs have attracted the attention of researchers in light of their important role as disease regulators and valuable diagnostic biomarkers [24]. Also, a series of circRNAs have been uncovered to be associated with the development of tumors, including BC [25]. Yang et al. revealed that circRNA circAGFG1 regulated the miR-195-5p/CCNE axis in triple-negative BC, which expedited cancer cell mobility, proliferation, and invasion [26]. A study pointed out that circRNA circ_0006528 contributed to BC progression through activating the MAPK/ERK pathway via targeting miR-7-5p [27]. Another study has demonstrated that circRNA circ_0136666 elevation facilitated BC progression by upregulating CDK6 via sponging miR-1299 [28]. In the current study, circ_SETD2 was downregulated in BC tissues and cells. Moreover, circ_SETD2 introduction curbed tumor growth *in vivo* and repressed cell cycle progression, proliferation, migration, and invasion, and facilitated apoptosis of BC cells *in vitro*. Afzali et al. discovered that circ_SETD2 was a differentially expressed gene that was downregulated in luminal A subtypes and triple-negative BC through microarray analysis [10]. Our data suggested that circ_SETD2 acted as a suppressor in BC, and the role of circ_SETD2 in BC was firstly reported.

Increasing research studies have reported that circRNAs could serve as miRNA sponges to regulate miRNA expression in the cytoplasm [29,30]. In this study, we found that miR-155-5p acted as a target for circ_SETD2 in BC cells. In a study, it was exhibited that miR-155-5p contributed to the advancement of hepatocellular cancer via repressing PTEN expression [14]. Previous research demonstrated that exosomal miR-155-5p secreted by melanoma cells could accelerate angiogenesis [31]. Also, miR-155-5p could promote cell invasion, migration, and growth in cervical cancer cells [13]. Herein, enhanced miR-155-5p expression overturned the effects of circ_SETD2 introduction on cell cycle progression, proliferation, apoptosis, migration, and invasion of BC cells. Besides, Wang et al. claimed that miR-155-5p upregulation decreased bufalin-induced apoptosis and accelerated proliferation of triple-negative BC cells [16]. And 3B (a new photosensitizer) could suppress tumor growth *in vivo* and repress miR-155-5p expression in BC cells *in vitro* [32]. Therefore, we inferred that circ_SETD2 inhibited the progression of BC via downregulating miR-155-5p. In addition, miR-155-5p was revealed to exert a suppressive role in lung adenocarcinoma [33] and gastric cancer [15], which might be related to the specificity of tumor tissue.

Additionally, we discovered that SCUBE2 acted as a target for miR-155-5p. It was reported that SCUBE2 enhancement constrained the invasion, migration, and proliferation of glioma cells [20]. Moreover, the poor prognosis of gastric cancer patients was associated with SCUBE2 downregulation [34]. SCUBE2 has been revealed to exert vital roles in inhibiting the invasion and mobility of BC cells [21]. Also, SCUBE2 overexpression constrained BC growth *in vivo* and repressed the proliferation of BC cells *in vitro* [35]. In our study, SCUBE2 overexpression curbed cell cycle progression, proliferation, migration, and invasion and promoted apoptosis of BC cells. SCUBE2 inhibition reversed miR-155-5p silencing-mediated effects on the malignant behavior of BC cells. In addition, the upregulation of SCUBE2 in BC cells mediated by circ_SETD2 introduction was restored by miR-155-5p overexpression. Therefore, we concluded that circ_SETD2 inhibited the malignant behavior of BC cells via upregulating SCUBE2 via miR-155-5p.

In all, circ_SETD2 overexpression repressed BC progression via regulating the miR-155-5p/SCUBE2 axis, indicating that circ_SETD2 might be a potential target for BC treatment. This study provided a novel strategy for improving the prognosis of BC patients.
